# Validol in Dipsomania

**Published:** 1911-09-23

**Authors:** Theo M. Kendall

**Affiliations:** formerly Surgeon St. Vincent's Hospital, Sydney, N.S.W. 37 Walbrook, E.C.


					YALIDOL IN DIPSOMANIA.
To the Editor of The Hospital.
Sir,?Some months ago I drew the attention of the pro-
fession to the value of a combination of menthol and vale-
rianic acid in the treatment of sea-sickness and train-
eicknese. Further experiments with this preparation,
which is sold under the name of " Validol," have demon-
strated to my satisfaction that it is a very valuable agent
in combating the evil effects of alcoholic indulgence and
of the drug habit. I find that it relieves all those nauseat-
ing feelings and cravings which are the concomitants of
alcoholic excess, gradually abolishes all the desire for
alcohol, and minimises the longing for drugs.
I have also found it useful in alleviating the effects of
chloroform inhalation, but as yet my experience in this
direction is too limited to pronounce a definite judgment.
I will be glad to hear if others have tried this preparation
for euch troubles.?I am, faithfully yours,
Theo M.
Kendall, formerly Surgeon St. Vincent's Hospital, Sydney,
N.S.W.
37 Walbrook, E.C.,
September 16.

				

